# The Value of Serum Prealbumin in the Diagnosis and Therapeutic Response of Tuberculosis: A Retrospective Study

**DOI:** 10.1371/journal.pone.0079940

**Published:** 2013-11-19

**Authors:** Hu Luo, Bingjing Zhu, Liang Gong, Jingxiang Yang, Yongyuan Jiang, Xiangdong Zhou

**Affiliations:** Department of Respiratory Medicine, the First Affiliated Hospital of Third Military Medical University, Chongqing, China; Glaxo Smith Kline, Denmark

## Abstract

**Objective:**

The aim of this study was to examine serum prealbumin (PA) levels in patients with tuberculosis and lung cancer, and to evaluate the correlations of serum PA levels with clinicopathological characteristics.

**Method:**

Total 760 patients were included in the study: 320 patients with tuberculosis, 320 patients with lung cancer, and 120 healthy subjects. Serum PA was detected using a biochemical analyzer to determine the value of serum PA in the diagnosis and therapeutic response of tuberculosis.

**Results:**

Compared to lung cancer and healthy individuals, TB patients were more frequent in suffering from low serum PA (75.0% vs.30.9% vs.6.7%,*P<*0.01), and the serum PA levels of TB patients were significantly reduced (137.5±42.4 mg/L vs. 183.5±49.1 mg/L vs. 240.0±43.9 mg/L, *P<*0.01). Among various clinical characteristics, type (with pleuritis), age (≥60), ESR (>20 mm/h) and smoking status (≥20 pack×years) were associated with low serum PA levels of TB patients, while ECOG performance status (≥2) was associated with low serum PA levels of lung cancer patients. The change of serum PA levels was in accordance with the therapeutic effects of anti-TB drugs, which might present a valuable and objective indicator for monitoring the therapeutic effects of TB drugs on TB patients.

**Conclusion:**

Low serum prealbumin levels are very common in TB patients and can be served as a potential indicator for differential diagnosis of lung cancer and monitoring the therapeutic effects of TB drugs.

## Background

Tuberculosis(TB) constitutes a substantial health problem in the developing countries [1], particularly in the face of the human immunodeficiency virus (HIV) co-pandemic [2] and the increasing incident of drug-resistant *Mycobacterium tuberculosis* strains [3]. According to a World Health Organization report in 2011 [4], there were an estimated 8.7 million new cases of TB (13% co-infected with HIV) and 1.4 million people died from TB, including almost 1 million deaths among HIV-negative individuals and 430,000 among people who were HIV-positive. China, second to India, was one of the 22 high TB burden countries which alone accounted for 12% of global newly cases (0.9 million–1.1 million).There were about 140 million TB patients in China till 2011, among which 26% patients were multi-drug resistant TB (MDR-TB).

Early diagnosis and timely standardized treatment are vital for the control of TB. However, the early differential diagnosis of TB from lung cancer still encounters a big problem due to lack of special symptoms and low sputum-positive rate in the patients [5]. Although new rapid molecular test including Xpert MTB/RIF [6] has the potential to substantially improve and accelerate the diagnosis of TB, very few patients with TB can receive this kind of test because these new laboratory techniques have not been widely applied. Besides, it is hard for physicians to decide the right time to stop anti-TB drugs due to lack of generally accepted and feasible indicators. Therefore, discovering new convenient, feasible marker(s) for the diagnosis of TB and assessment of the efficacy of anti-TB drugs will be of great importance in clinical practice.

Prealbumin (PA) is a kind of plasma protein synthesized by the hepatocytes with a short biological half-life (∼1.9 d). The main physiological function of PA is transportation of thyroxine and vitamin A to enhance the body's immunity by promoting the maturation of lymphocytes [7]. Studies have suggested that the concentration of PA is sensitive and specific for the diagnosis of patients with liver cell damage [8], [9]. In addition, it has also been widely accepted that PA, which is more sensitive than serum albumin, can be a useful indicator in assessing nutritional status and monitoring the effect of nutritional support [10], [11], [12], [13].

Emerging evidence has been demonstrating that the presence of nutritional imbalance is very common in the patient with tuberculosis [14], [15],but the level of serum PA in those patients has not been examined. Thus, the purpose of this study was to examine the relationship between serum PA concentrations and various clinical features of TB patients in order to address whether serum PA concentrations could be an indicator in the early differential diagnosis of TB from lung cancer. Furthermore, we also monitored the dynamic changes of serum PA level in the patients during TB treatment to find out whether it could be useful to reflect the disease control or improvement during TB treatment.

## Materials and Methods

### Ethics Statement

The study complies with the standards of the Declaration of Helsinki and current ethical guidelines and was approved by Ethic Committee of Southwest Hospital (First Affiliated Hospital of Third Military Medical University), the Third Military Medical University. The Ethic Committee approved that informed consent was not required because data were going to be collected retrospectively and analyzed anonymously.

### Sample Population

A total of 320 newly confirmed TB patients with avaliable clinical datas were taken into our study, and 320 patients with lung cancer and 120 healthy individuals visiting the First Affiliated Hospital of Third Military Medical University (also named Southwest Hospital, the biggest teaching hospital in Chongqing, China) during the same period (from January 2011 till December 2012) were randomly chosen from our database. All the patients met a number of criteria (Figure S1). Clinical data (including age, gender, body mass index(BMI), smoking status, results of liver function and serum PA test) of all patients should be complete. They had not accepted anti-TB or anti-cancer therapy at least one month before the study. None of the subjects showed signs of acute respiratory infection including throat discomfort or sore throat, sneezing, runny nose, nasal congestion, hyperemia or edema in nose, pharynx and larynx at least two weeks before the investigations. Subjects were also excluded if they had a history of another malignancy or other diseases associated with malnutrition such as chronic diarrhea, liver cirrhosis, malabsorption syndrome, as these diseases may influence serum PA levels. All these tuberculosis patients were comprehensively diagnosed by the experienced physicians based on symptoms (including mild fever, cough, sputum, hemoptysis, night sweats, fatigue and so on), tubercle bacillus in sputum, tuberculin test (PPD), tuberculosis T cells spot experiment (T-SPOT) and biopsy, while all lung cancer patients were all pathologically diagnosed through bronchoscope or CT-guided percutaneous lung puncture. The clinical characteristics of the study population were described in Table 1. TB patients accepted anti-TB therapy according the Guidelines for the Prevention and Management of Tuberculosis in China (2008 edition). All data were collected by investigators from the electronic medical records system.

**Table 1:** Clinical Characteristics of the Study Population.

Variable	Patients withTuberculosis (N = 320)	Patients withLung cancer (N = 320)	Healthy Individuals(N = 120)
Gender, n (%)			
Male	180(56.3)	201(62.8)	64(53.3)
Female	140(43.7)	119(37.2)	56(46.7)
Age, mean±SEM, (years)	41.5±1.0	58.4±1.1*	43.7±1.1
BMI, mean±SEM, (kg/m^2^)	21.7±0.1	22.3±0.2	22.1±0.2
Hepatitis B, n (%)			
Yes	14(4.4)	17(5.3)	8(6.7)
Transaminase elevation, n (%)			
Yes	7(2.2)	5(1.6)	2(1.7)
Smoking status, n (%)			
Never-smokers	158(49.4)	126(39.4)	61(50.8)
Ex-smokers	11(3.4)	15(4.9)	5(4.2)
Smokers	151(47.2)	179(55.9)	54(45)
PA decreased, n(%)	240(75)***	99(30.9)	8(6.7)
PA level, mean±SD (mg/L)	137.5±42.4**	183.5±49.1	240.0±43.9

AST≥40 IU/L or ALT≥40 IU/L were defined as Transaminase elevation;Serum PA levels≤170 mg/L were defined as PA decreased.

* *P<*0.05, compared to both TB and healthy individuals groups.

*** *P<*0.001, compared to both lung cancer patients and health individuals groups.

** *P<*0.01, compared to both lung cancer patients and health individuals groups.

### Detection of Hepatitis Virus B Markers

Peripheral venous blood samples were taken from the patients before they received anti-TB or anti-cancer therapy and analyzed by using an automatic ELISA analyzer (TECAN Group Ltd., Switzerland).

### Liver Function Tests

Peripheral venous blood samples were taken before receiving anti-TB or anti-cancer therapy and analyzed by using an automatic biochemical analyzer Olympus AU2700. Aspartate aminotransferase(AST)≥40 IU/L or alanine aminotransferase(ALT)≥40 IU/L were defined as Transaminase elevation.

### Smoking Status

The study subjects were divided into 3 categories according to their smoking status: smokers who are currently smoking with a smoking history or more than 10 pack-years, ex-smokers who has a smoking history of more than 10 pack-years but stop smoking more than 2 years before the study, and never-smokers who have never smoked. To calculate pack-years of smoking, the total number of years of smoking was multiplied by the average number of cigarettes smoked per day and divided by 20 (years × cigarettes per day/20).

### Serum Preabulmin Measurement

Peripheral venous blood samples were taken from patients in the morning before meal. Serum PA levels were measured using an automatic biochemical analyzer Olympus AU2700 (Olympus, Japan). Serum PA levels≤170 mg/L were defined as abnormal. PA was routinely measured monthly when these patients come back for return visit.

### Statistical Data Analysis

Statistical data analysis was performed using the statistical SPSS for Windows 17.0 software package (SPSS, Chicago, USA). The data are presented as the mean ± SD with ranges. The categorical data were compared using *χ* ^2^ test. Differences among all the study groups (more than two groups) were evaluated using the one-way ANOVA. Differences between two groups were evaluated using the Student’s t test. Statistical significance was set at *P<*0.05.

## Results

### Serum PA Levels in the TB Group are Significantly Lower than Lung Cancer and Healthy Groups

320 newly confirmed TB patients, 320 patients with lung cancer and 120 healthy individuals were finally taken into our study.The clinical characteristics of the study population were described in Table 1. No significant differences in gender, BMI, smoking status, smoking intensity and hepatitisB except age were found among all the investigated groups.

Reduced serum PA levels (*<*170 mg/L) were observed more frequent in the TB patients than in the patients with lung cancer or in the healthy individuals group (*P<*0.05, seen in Table 1). The lowest serum PA levels were found in the TB group compared with the patients with lung cancer or healthy group (*P<*0.01, seen in Table 1). It is worth mentioning that the serum PA levels in the patients with lung cancer remained in the normal ranges (170 ∼ 420 mg/L) although they were significantly lower than those of healthy individuals.

### Influencing Factors of PA Decrease in Patients with TB or Lung Cancer

**Figure 1:**
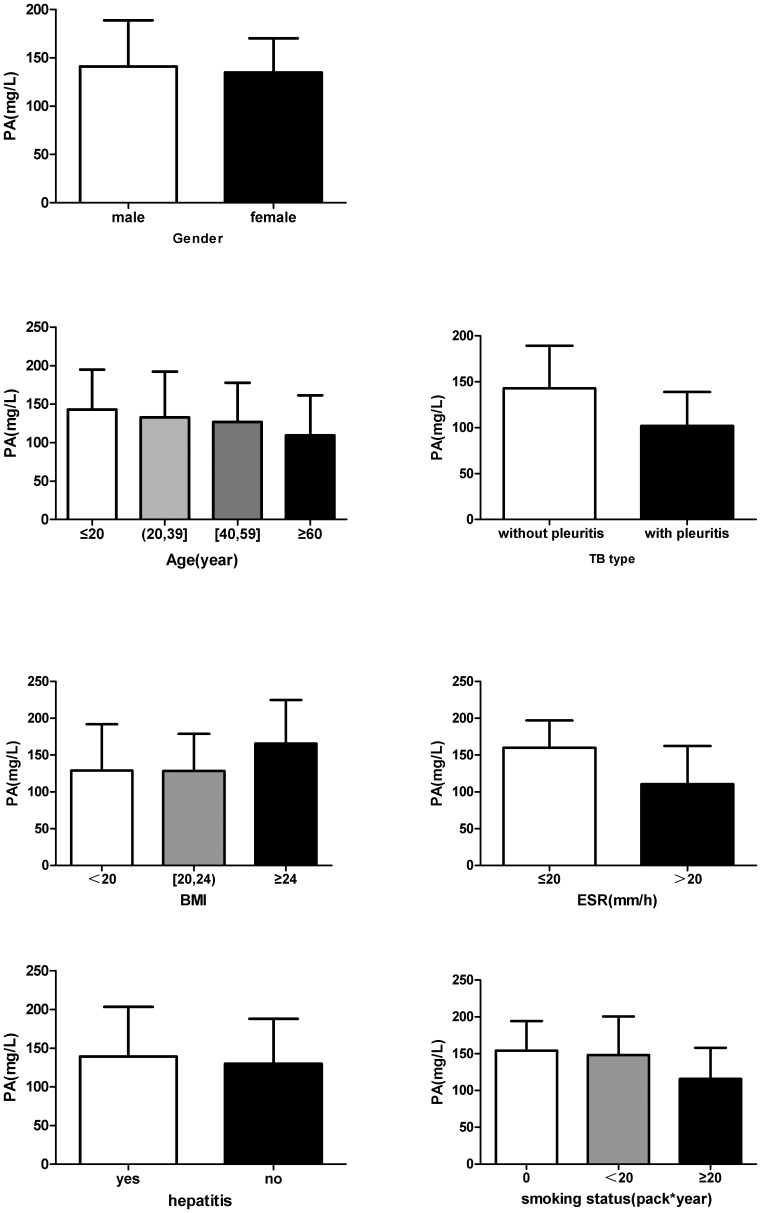
The serum PA levels in different subgroups of TB patients. There were significant differences in the serum PA levels between following subgroups: pleuritis and lung tuberculosis, TB patients with higher ESR (≥20 mm/h) and normal ESR (*<*20 mm/h), TB patients with higher smoking status (≥20 year×pack) (*P<*0.01).

As we have found that PA decrease widely existed (75%) in TB patients before they accepted anti-TB treatment, we further explored the factors which may be associated with low serum PA. The results (Table 2) showed that TB type age, erythrocyte sedimentation rate (ESR) and smoking status could be influencing factors which may lead to TB patients more prone to suffering from serum PA decrease. Furthermore, in tuberculosis group, we found that there were significant differences in the serum PA levels between the following subgroups (Figure 1): pleuritis and lung tuberculosis (105.1±30.7 mg/L vs. 146.7±40.8 mg/L, *P<*0.01), TB patients with higher ESR and normal ESR (122.5±40.4 mg/L vs. 160.3±37.2 mg/L, *P<*0.01), and TB patients with different smoking status (121.9±44.7 mg/L vs. 148.1±51.6 mg/L or 154.2±46.5 mg/L, *P<*0.01).

**Table 2:** Influencing factors of PA decrease in patients with TB.

Variables	PA decreased(n/N)	%	*χ* ^2^	(*P* value)
Gender				
Male	132/180	73.3	0.610	0.435
Female	108/140	77.1		
Age				
*<*20	15/21	71.4	12.363	0.006** ^a^
20–39	82/123	66.7		
40–59	109/139	78.4		
≥60	34/37	91.9		
Type				
Lung TB	178/249	71.5	7.391	0.007**
with pleuritis	62/71	87.3		
BMI				
*<*19	49/60	81.7	0.284	0.241
19–23.9	181/244	74.2		
≥24	10/16	62.5		
ESR(mm/h)				
>20	153/190	85.8	7.618	0.006**
≤20	87/130	59.2		
Hepatitis B				
yes	9/14	64.3	0.896	0.334
no	231/306	75.5		
Smoking status (male only)				
0	24/40	60.0	14.326	0.001** ^b^
*<*20	28/43	65.1		
≥20	84/97	86.6		

Serum PA levels≤170 mg/L were defined as PA decreased;

Patients were divided into two types:lung TB(pulmonary tuberculosis patients without pleuritis) and with pleuritis(incluing both pulmonary tuberculosis patients with pleuritis and simple pleuritis patients).

** *P<*0.01.a: age≥60 compared to other subgroups; b: smoking status≥20 compared to other subgroups.

Similarly, there were no significant differences in the serum PA levels among the major lung cancer histological types: adenocarcinoma, squamous cell carcinoma and small cell lung carcinoma (184.4±47.5 mg/L vs.188.1±49.0 mg/L vs.180.2±38.0 mg/L, *P*>0.05) as well as non-small cell lung cancer and small cell lung cancer groups (187.6±48.1 mg/L vs. 180.2±38.0 mg/L, *P*>0.05). Serum PA levels were significantly higher in the patients with low Zubrod performance status (ZPS) (198.4±49.8 mg/L vs. 158.5±47.8 mg/L, *P*<0.01). However, no significant difference in serum PA levels was found between early stage and advanced lung cancer patients (177.1±32.6 mg/L vs.185.3±49.4 mg/L, *P*>0.05).

Besides, some studies have declared that low serum PA may present a predict indictor of early liver damage(mainy reflected by elevated transaminase) caused by anti-TB drugs. In our study, patients were divided into analytical groups with <170 mg/L and ≥170 mg/L and we examined the relationship between serum PA concentrations and liver damage (all liver damage during the anti-TB were included) after anti-TB drugs. However, no significant difference in liver damage was found between the PA-decrease and PA-normal subgroups (*χ* ^2^ = 0.471, *P* = 0.389), implying that PA decrease may not be a risk factor of liver damage.

### Changes of Serum PA Levels are in Accordance with the Therapeutic Effects of Anti-TB Drugs

It was widely accepted that serum PA could be a useful indicator in evaluating nutritional status and monitoring the effect of nutritional support. Patients with tuberculosis were thought suffered from nutritional imbalance [16], [17]. However, the role of PA in assessing the therapeutic effects during the anti-TB treatment has not been well elucidated. Thus, we examined PA levels at different time point (1^st^ month, 3^rd^ month, 6^th^ month, 9^th^ month and 12^th^ month) after patients accepted anti-TB treatment. As a control, we also examined the change of serum PA levels from both drug-resistant TB patients and lung cancer patients at the same time point as described above.

**Figure 2:**
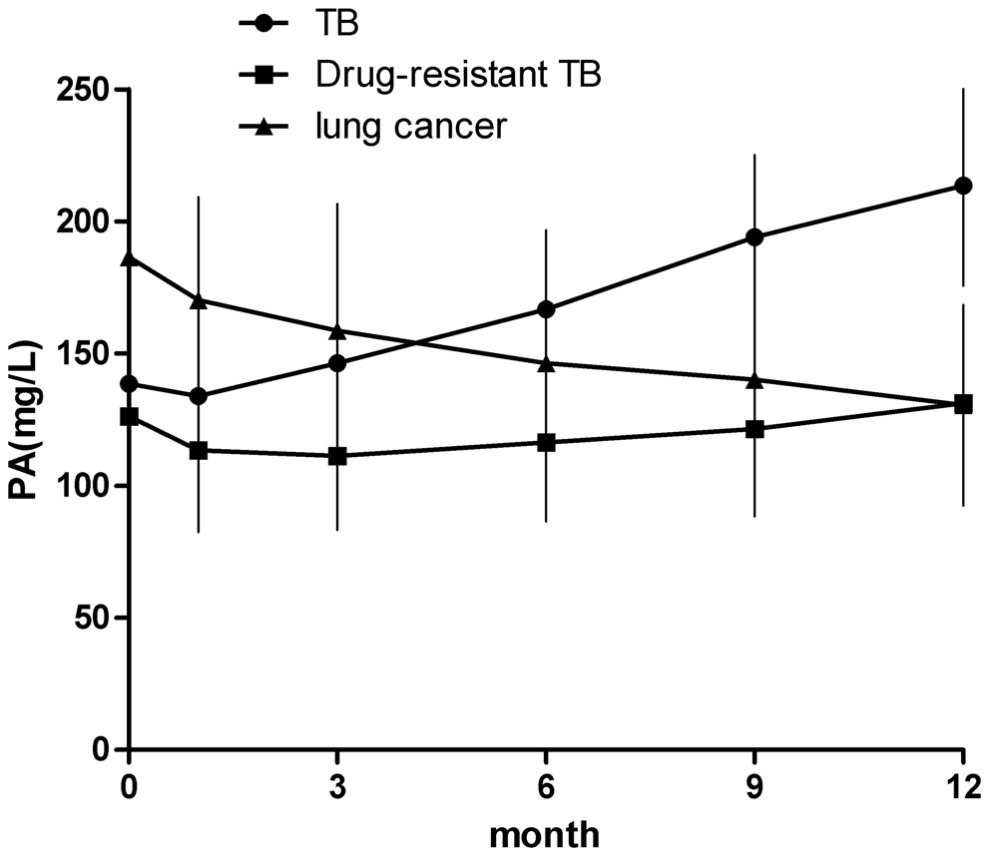
The change of serum PA levels in different group of patients. Serum PA levels slowly elevated after the TB patients accepted anti-TB drugs. Nine months after use of anti-TB drugs, the average of serum PA levels (194.1±29.2 mg/L) among these TB patients significantly rised to normal range. However, the serum PA levels of drug-resistant TB patients remained at a low level state. The serum PA levels in lung cancer patients were slowly reduced after chemotherapy.

As showed in Figure 2, after TB patients accepted anti-TB drugs, the serum PA levels slowly elevated. 9 months after use of anti-TB drugs, the average of serum PA levels (194.1±29.2 mg/L) among these TB patients significantly reached to normal range. However, for these drug-resistant TB patients (48/320, 15%), the serum PA sustained at a low level state. Interestingly, the average serum PA levels in lung cancer remained normal (186.5±49.1 mg/L) at the time of diagnosis, but slowly reduced along with chemotherapy treatment. After 4 cycles (about 3 months) of chemotherapy finished, the PA levels among these lung cancer patients were reduced to a low status (158.7±47.7 mg/L) and remained unchanged.

## Discussion

This study was designed to evaluate serum PA levels in the patients with tuberculosis and lung cancer. The key observations made in this study can be summarized as follows: 1) Compared to lung cancer and healthy individuals, TB patients were more frequent in suffering from low serum PA, and the serum PA levels of TB patients were significantly reduced; 2) among various clinical characteristics, type (with pleuritis), age (≥60), ESR (>20 mm/h) and smoking status (≥20 pack×years) may be associated with low serum PA levels of TB patients, while ZPS (≥2) may be associated with low serum PA levels of the patients with lung cancer; and 3) the change of serum PA levels could be a useful indicator in monitoring the disease improvement of TB patients.

Despite various control efforts, tuberculosis still remains a major public health challenge in much of the developing and transitioning world, with an estimated 8.7 million people of global population latently infected, and about 1.4 million deaths in 2011**.** The early diagnosis and timely standard treatment are extremely important for the ideal outcome of tuberculosis [18], [19]. However, tuberculosis sometimes is difficult to be distinguished from other lung disease [20], [21], [22], especially lung cancer [23], [24], due to lack of specific symptoms and biomarkers. Besides, irregular treatments, including stopping anti-TB drugs too early, were the major courses of drug resistance and recurrence [25], [26]. However, sometimes it was difficult for physicians to decide the right time to stop anti-TB drugs because of the following reasons: 1) physician should be more careful due to the high prevalence of MDR-TB in China [27], and 2) the standards for drug withdrawal are not so objective and highly depended on the physician’s experience [28].

In recent years, the serum PA levels have been reemphasized by extending its clinical use to the severity of disease, disease progression, and prognosis [29], [30]. As in many previous studies, serum PA was thought superior to albumin in assessing an individual’s recent nutritional intake and current nutritional state [10], [11], [31] as it has a shorter biological half-life. Furthermore, serum PA insufficiency was also reported relating to higher recurrence risk after surgery [32], liver cirrhosis [9], [33], inflammatory processes [34], wound healing [35], kidney injury [36], [37], [38] and liver insufficiency [39]. As liver damage was a common side effect in the process of anti-TB, serum PA was considered to be a sensitive indictor to predict early liver injury caused by anti-TB drugs. However, in clinical work, we noticed that lots of TB patients suffered from serum PA decrease before they accepted anti-TB therapy. In this study, we confirmed that decreased serum PA widely existed (75.0%) in TB patients before they used anti-TB drugs, and no significant difference in the incidence of liver damage was found between the PA-decreased and PA-normal subgroups, suggesting that serum PA levels might not a predictor of early liver damage.

Lung cancer has been the leading cause of cancer-related death all over the word. In 2008 of China, both the incidence (18.5%) and mortality (23.1%) of lung cancer have ranked the first among all kinds of malignancies [40]. Due to symptomatic and radiological similarities, a large number of TB patients were initially hard to be distinguished from lung cancer. To our knowledge, the present study was the first to demonstrate that the frequency of low serum PA was significantly higher in TB patients compared with lung cancer patients and healthy individuals, and PA levels among TB patients was significantly lower than those lung cancer patients. Those results suggest that serum PA levels could be valuable in the differential diagnosis between TB and lung cancer, although the sensitivity and specificity need to be further investigated. We also found that type (with pleuritis), age (≥60), ESR (>20 mm/h) and smoking status (≥20 pack×years) may be associated with low serum PA levels of TB patients, while the ZPS (≥2) was associated with low serum PA levels of lung cancer patients. These results revealed that low serum PA levels may be in accordance with the activity and severity of both TB and lung cancer. Since serum PA is a good marker of nutritional status, we believe that the improved nutritional status may be of great importance during the treatment of both TB and lung cancer.

Furthermore, serum PA levels in most TB patients elevated slowly after using anti-TB drugs. About 9 months later, serum PA levels of 80.6% TB patients (258/320) have elevated to the normal range and the average was 194.1±29.2 mg/L. However, serum PA levels of those drug-resistant TB patients sustained at a low status, and no significant increase even after 12 months. These results indicate that the change of serum PA levels are in accordance with the therapeutic effects of anti-TB drugs, which might present a good and objective marker in monitoring the treatment effects for most TB patients and warning the possibility of drug-resistance if the PA levels remain at a low state. In contrast, as serum PA levels among lung cancer patients were slowly reduced after chemotherapy [32], we speculated that sustained reductions of serum PA levels might also predict a poor outcome [41], [42]. However, due to its short half-life, the decrease in prealbumin concentration observed in patients during chemotherapy for lung cancer could also be the result of treatment associated anorexia and/or other adverse effects.So, prospective study should be well designed to elucidate the relationship between serum PA levels and outcomes of lung cancer patients in the near future.

It is necessary to address a few limitations to our study. The major limitation of the study is that it used retrospective methods of data collection. Besides, the baseline including age and smoking status among 3 groups were not well matched because the median age of patients with lung cancer was much older than those of TB patients. However, as the results in Table 1 suggested that advanced age (≥60) presented a potential risk factor for these TB patients in suffering from low serum PA, the findings of that the older patients in lung cancer groups had a significantly higher serum PA level strengthened the point that TB patients were more prone to undergoing low serum PA.

## Conclusions

In summary, we demonstrated that serum PA levels were significantly reduced in the TB patients compared with the patients with lung cancer and healthy subjects, and the change of serum PA levels could be an objective marker in monitoring the therapeutic effects of TB drugs and warning the possibility of drug-resistance if the PA levels sustained a low state. Furthermore, as serum PA levels reduced after chemotherapy and proved to be associated with poor performance status, the value of serum PA levels in prognosis of lung cancer is worthy of further investigating.

## Supporting Information

Figure S1 **The flow chart of the study.** Patients met a number of criterias were chosen into our study.
(DOC)
