# Supplementary material for: The Value of Serum Prealbumin in the Diagnosis and Therapeutic Response of Tuberculosis: A Retrospective Study
Source: PLoS One. 2013 Nov 19;8(11):e79940. doi: 10.1371/journal.pone.0079940 (PMC3833965; doi:10.1371/journal.pone.0079940)
Supplement: Figure S1 — The flow chart of the study. Patients met a number of criterias were chosen into our study. (DOC) [file pone.0079940.s001.doc]

TB group (N=320)

Project of a retrospective study has been launched, then patients were chosen from the database

**Exclude criteria:**

1. Incomplete of clinical data
2. Accepted anti-TB or anti-cancer drugs in 1 month
3. Suffered from acute respiratory infection in 2 weeks
4. Accompanied by other malnutrition diseases
5. Lung cancer patients with another malignance

Lung cancer group (N=320)

Healthy individual group (N=120)

Data collection General characteristics: gender/age/BMI/smoking status/liver function… Serum PA: before treatment and during the treatment

Data analysis and discussion about results

**Figure S1** the flow chart of the study
